# Idiopathic Mesenteric Phlebosclerosis: A Single-Institute Experience in Taiwan

**DOI:** 10.5152/tjg.2023.22335

**Published:** 2023-05-01

**Authors:** Jen-Wei Chou, Chia-Hsi Chang, Yi-Hua Wu, Kai-Chih Chang, Ken-Sheng Cheng, Po-Ju Huang

**Affiliations:** 1 China Medical University Faculty of Medicine, Taichung, Taiwan; 2 Department of Internal Medicine, Center for Digestive Medicine, China Medical University Hospital, Taichung, Taiwan; 3 Taiwan Association for the Study of Small Intestinal Diseases (TASSID), Touyuan, Taiwan; 4 Division of Gastroenterology and Hepatology, Department of Internal Medicine, Asia University Hospital, Taichung, Taiwan

**Keywords:** Computed tomography, idiopathic mesenteric phlebosclerosis, ischemic bowel disease, plain abdominal radiography, phlebosclerotic colitis

## Abstract

**Background:**

Idiopathic mesenteric phlebosclerosis is a rare condition with unclear pathogenesis. This study aimed to investigate the clinical features, diagnostic modalities, treatments, and outcomes of idiopathic mesenteric phlebosclerosis patients in Taiwan.

**Methods:**

Idiopathic mesenteric phlebosclerosis patients diagnosed by the typical characteristic of tree-like mesenteric venous calcifications on plain abdominal radiography or computed tomography between January 1992 and July 2021 were retrospectively analyzed.

**Results:**

: Totally, 36 idiopathic mesenteric phlebosclerosis patients were enrolled (50% females; mean age, 61.6 years). Among the included patients, 26 (72.2%) and 10 (27.7%) were symptomatic and asymptomatic, respectively. Abdominal pain (61.1%) accounted for the majority of all symptoms, followed by fever, diarrhea, and bloody stools. Our results showed that 83.3% of patients had at least 1 risk factor, whereas 16.6% of patients had none. Moreover, among the included patients, 36.1%, 44.4%, 50.0%, 38.8%, and 8.3% had cardiovascular disease, chronic renal disease, cancer, chronic liver disease, and diabetes mellitus, respectively. Our findings showed 94.4% of patients were diagnosed via abdominal computed tomography and plain abdominal radiography, whereas 5.6% of patients were diagnosed via plain abdominal radiography. The ascending colon was the most commonly involved site (100%). Our findings showed that 91.6% of patients experienced good recovery after conservative treatment, except for the 3 who died of sepsis and respiratory failure. By contrast, 8.3% of idiopathic mesenteric phlebosclerosis patients underwent colectomy. The average follow-up duration was 62.5 months.

**Conclusions:**

Idiopathic mesenteric phlebosclerosis remains a rare disease in Taiwan. Plain abdominal radiography and computed tomography can be utilized for establishing a definite diagnosis. Conservative treatment is usually adequate for most patients, with surgical treatment only indicated for severe cases.

**Main Points**

- Idiopathic mesenteric phlebosclerosis (IMP) is a rare condition with an unclear pathogenesis.
- The diagnosis of IMP was based on the typical characteristic of tree-like mesenteric venous calcifications on plain abdominal radiography or computed tomography.
- Conservative treatment is usually adequate for most patients with IMP. On the contrary, surgical treatment is only indicated for severe cases.

## Introduction

Etiologies of ischemic bowel disease include thrombosis or embolism of mesenteric vessels, bleeding disorders, drugs, trauma, amyloidosis, shock, and iatrogenic conditions.^1^ Idiopathic mesenteric phlebosclerosis (IMP), originally described in 1989 by Iwashita et al^2^, is an exceedingly rare type of ischemic bowel disease characterized by colonic wall thickening associated with fibrosis, hyalinization, and calcification of the affected veins and submucosa. While Yao et al^3^ initially named this disease “phlebosclerotic colitis” in 2000, Iwashita et al^4^ subsequently coined the term “idiopathic mesenteric phlebosclerosis” in 2003. A review of the literature showed that the IMP was most prevalent among the Japanese population.^1-5^ Moreover, a few cases of IMP had been reported from Asian countries other than Japan, including Taiwan, Hong Kong, and Korea.^6-10^ In contrast, this disease was rarely reported in Western countries.^11,12^

Despite the current lack of a definite consensus on the treatment of IMP, surgery has been the mainstay approach, according to available studies. Thus, the present study aimed to investigate the clinical features, diagnostic methods, and treatment of IMP in a single institute located in middle Taiwan.

## Materials and Methods

The study was approved by local the Ethics Committee of China Medical University Hospital.

### Study Population

From January 1992 to July 2021, medical chart records of patients diagnosed with IMP at China Medical University Hospital, a tertiary referral hospital, in middle Taiwan were retrospectively collected and reviewed. The diagnostic criteria were based on the typical imaging findings on plain abdominal radiography or computed tomography (CT). Plain abdominal radiography and abdominal CT scans demonstrated typical tree-like linear venous calcifications along the wall of the colon or small bowel. Age, sex, symptoms, comorbidities, diagnostic modalities, treatment methods, and clinical outcomes of all patients were analyzed. The informed consent was not necessary due to the retrospective study.

### Statistical Analysis

The results were expressed as the mean ± standard deviation (SD), ranges, median, or percentages. Continuous variables were represented as the mean ± SD unless otherwise stated. Categorical variables were represented as frequency analysis, n (%). All statistical analyses were performed using the International Business Machines Statistical Package for the Social Sciences (SPSS) Statistics for Windows, version 19 (IBM Corp., Armonk, NY, USA).

## Results

A total of 36 patients diagnosed with IMP were enrolled in the present study, and their demographic characteristics are summarized in Table 1. Among the included patients, 18 were female and 18 were male, with a female-to-male ratio of 1:1. The mean age of all patients was 61.6 years (range, 43-85 years). Moreover, 26 (72.2%; 26/36) and 10 (27.8%, 10/36) patients were symptomatic and asymptomatic, respectively. Analysis of symptoms showed that abdominal pain accounted for the most common symptom, appearing in 22 patients (61.1%, 22/36), followed by diarrhea in 4 patients (11.1%, 4/36), fever in 3 patients (8.3%, 3/36), and bloody stool in 1 patient (2.7%, 1/36). In terms of comorbidities, 30 patients (83.3%, 29/36) had at least 1 risk factor, whereas only 6 patients (16.7%, 6/36) had no demonstrable major systemic disease upon presentation. Subgroup analysis of comorbidities showed that 13 patients (36.1%, 13/36) had cardiovascular disease, including hypertension, heart failure, stroke, coronary heart disease, and valvular heart disease; 16 patients (44.4%, 16/36) had renal disease, including chronic kidney disease and end-stage renal disease with or without hemodialysis; 14 patients (38.8%, 14/36) had chronic liver disease, including chronic hepatitis B, chronic hepatitis C, and liver cirrhosis; 18 patients (50.0%, 18/36) had a cancer history, including transitional cell carcinoma (9 patients), hepatocellular carcinoma (6 patients), hepatic angiosarcoma (1 patient), renal cell carcinoma (1 patient), and gastric cancer (1 patient); and 3 patients (8.3%, 3/36) had diabetes mellitus. Regarding the usage of Chinese herbal medicines, our findings showed that identified 13 patients (36.1%, 13/36) had a long-term usage history of Chinese herbal medicines (symptomatic-9; asymptomatic-4).

The diagnostic modalities, involving bowel segments, treatment methods, and clinical outcomes, of patients with IMP are detailed in Table 2. Regarding the diagnostic modalities, plain abdominal radiography was performed in 32 patients, among whom 25 (78.1%, 25/32) showed typical characteristics of linear tree-like calcifications along the involved bowel segments. Whereas abdominal CT was performed in 34 patients, among whom 34 (100.0%, 34/34) demonstrated venous calcifications in the mesenteric veins of the colon and small bowel, and edematous colonic wall thickening with surrounding fat stranding. Colonoscopy was performed only in 12 patients (33.3%, 12/36), which revealed dark purple mucosa, luminal narrowing, erosions, ulcers, and hyperemia. Barium enema and angiography were performed on those who revealed a loss of normal mucosal haustrations and vein dilatation along the vasa recta. Regarding the involved bowel segments, our findings showed that the ascending colon was the most commonly involved site in all patients (100%, 36/36), followed by the transverse colon (23/36, 63.8%), the descending colon (8.3%, 3/36), and the terminal ileum (2.7%, 1/36).

Among the included patients, 33 (91.7%, 33/36) received conservative treatment, whereas 3 (8.6%, 3/36) underwent emergency total colectomy or subtotal colectomy due to peritonitis severity and fever at presentation. Among those who received conservative treatment group, 10 asymptomatic patients received only follow-up, whereas the remaining 23 symptomatic patients received bowel rest, fluid supplement, and prophylactic antibiotics. Almost all patients who received conservative treatment had favorable outcomes with symptomatic relief, except for the 3 who died of sepsis within 3 months due to other severe medical diseases. Meanwhile, none of the 3 patients in the surgical treatment group died or developed complications after surgery.

## Discussion

Idiopathic mesenteric phlebosclerosis is a rare disorder that has usually been reported in Asian countries, especially in East Asia^.6-10^. Only a very few cases of IMP have been reported in Western countries, most of whom were from Asia.^11-12^ Thus, genetic susceptibility and region-specific lifestyles are likely involved in the pathogenesis of this disease based on the characteristics of geographic distribution. Previous studies have shown that IMP usually occurs in the elderly, with a slight female predominance. Yoshinaga et al^13^ reported 19 Japanese patients with IMP and found the mean age of onset was 59.1 years, with 63% of the cases being female. Moreover, a large case series by Guo et al^14^ involving 52 patients with IMP from PubMed found that the average age of all patients with IMP was 59.8 years, with 50% being men. After conducting a retrospective review of 25 patients with IMP at 2 affiliated hospitals in China, Chen et al^15^ found that the mean age of all patients with IMP was 63.5 years, with male predominance (92%). The present study, on the other hand, found that the mean age of IMP patients was 61.6 years, with 50% of the cases being female. However, Chang^16^ also reported a mean age of 46.2 years in 5 Taiwanese patients with IMP, which was younger compared to that in previous reports and the present study.

Despite the implications of some predisposing factors, the pathogenesis of IMP remains unclear. Kitamura et al^17^ initially speculated that the pathogenesis of IMP is associated with the Raynaud phenomenon, esophageal involvement, sclerodactyly, and telangiectasia syndrome. Subsequently, Losanoff et al^18^ proposed that natural aging or various pathologic processes were involved in the etiology of IMP. Furthermore, Iwashita et al^4^ considered IMP as an adaptive change in the venous wall to the prolonged increased venous blood pressure (e.g., right-sided heart failure or portal hypertension). In addition, Iwashita^2^ claimed that patients’ social and occupational histories and clinical laboratory data of the patients offer no information regarding the etiology. Kusanagi et al^19^ also considered portal hypertension as a possible etiology of this disease. After analyzing the clinical features and histological findings from 5 patients with IMP, Chang^16^ found that all patients had a history of regular ingestion of Chinese herbal medicines for 4-20 years. Thus, he proposed that toxins or biochemicals may play a predominant role in the pathogenesis of IMP rather than aging. Since the publication of the aforementioned findings, some authors had also described an association between herbal medicines and IMP.^20-22^ Recently, a large-scale nation-wide survey investigating the association between herbal medicines and IMP by Shimizu et al^23^ found that 70.4% of the 169 patients used herbal medicines containing sanshishi. Accordingly, the present study found that 36.1% of all patients had a history of using Chinese herbal medicines, whereas the other patients lacked accurate detailed information on their usage. However, we believe that we underestimated the usage of Chinese herbal medicines in our patients with IMP given the ubiquitous usage of Chinese herbs or herbal medicines in Taiwan and other Asian countries. Moreover, diabetes, dyslipidemia, liver cirrhosis, and autoimmune disorders have all been implicated as potential causes of IMP.^16^ In fact, the present study found that 83.3% of patients had risk factors, including cardiovascular disease, chronic renal disease, chronic hepatitis and cirrhosis, diabetes mellitus, and cancers. However, 16.6% of all patients had no major risk factors at presentation. Based on the foreminded results, we agree with Iwashita’s opinion that IMP occurs as an adaptive change in the venous wall to the prolonged and increased venous blood pressure. Moreover, IMP should be distinguished from mesenteric inflammatory veno-occlusive disease (MIVOD), which is another rare type of ischemic bowel disease histopathologically characterized by phlebitis and venulitis in the acute phase and myointimal hyperplasia, phlebosclerosis, and thrombosis in the late phase.^24,25^ Some authors suggested the possibility that IMP may be an extremely advanced form of post-inflammatory non-thrombotic occlusive venopathy or even end-stage MIVOD. However, none of the manifestations of MIVOD have been identified in IMP.

Patients with IMP may present with acute or chronic symptoms, which can manifest as abdominal pain, diarrhea, constipation, vomiting, fever, or bloody stool, depending on the disease severity. The present study showed that the majority of all patients (72.2%) presented with acute symptoms, which differed from the chronic symptoms of most patients (89%) from the Yoshinaga’s^13^ report. Moreover, all of our patients showed abdominal pain as the major symptom (61.1%), with diarrhea, fever, and hemorrhage having been rarely observed. Furthermore, some previous reports have shown that patients with IMP may be asymptomatic and discovered incidentally.^26^ Chen et al^15^, who conducted a retrospective study on 25 patients with IMP found that 12% of such patients had no obvious symptoms. A case series by Kato et al^27^ also reported that 10% of patients with IMP remained asymptomatic. Meanwhile, the present study found that 27.7% of the patients with IMP were asymptomatic.

Regarding the involvement of bowel segments, evidence has shown that IMP mainly affects the proximal colon, including the ileocecum, ascending colon, and transverse colon owing for several possible reasons. First, the absorption of water and partial digestion mainly occurs in the ileocecal region and ascending colon, and the round-trip movement of haustrum causes repeated absorption of sitotoxin in the ileocecal region and ascending colon. Second, the movement of food through the ascending colon goes against gravity, which prolongs the retention time in the right colon.^28^ However, the IMP can also involve the terminal ileum cecum and descending colon. Ikehata et al^29^ reported that lesions in 2 cases of IMP gradually extended to the distal colon over a period of 5 years. Moreover, the present study found that the ascending colon was the most commonly involved site, followed by the transverse colon, descending colon, and terminal ileum. Furthermore, the correlation between IMP and colon cancer remains unspecified. To date, only a handful of IMP cases with concomitant adenocarcinoma has been reported in the literature.^15,30^

**Figure 1.**
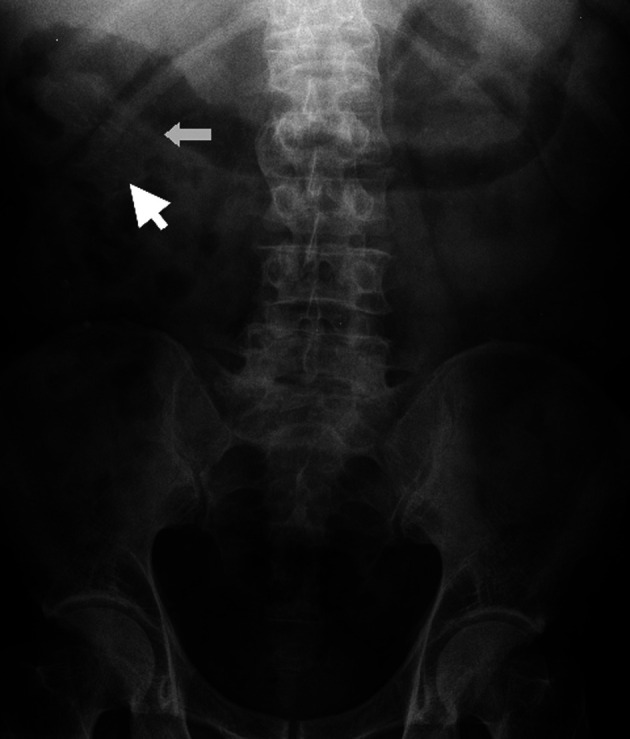
Abdominal plain radiography demonstrating multiple linear and thread-like calcifications at the site of the ascending colon (arrow).

**Figure 2.**
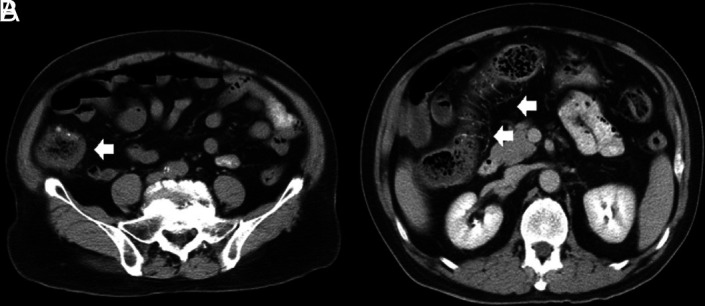
Abdominal computed tomography showing thickened colonic wall with marked nodular calcifications along the wall of the ascending colon (A, arrow) and transverse colon (B, arrows).

**Figure 3.**
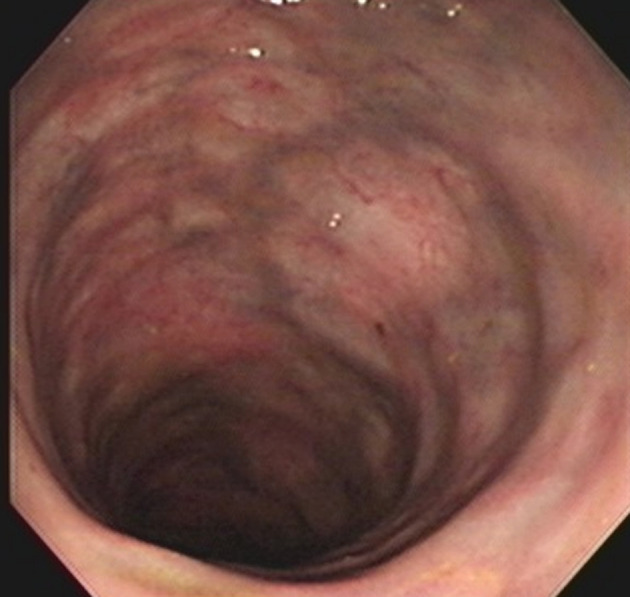
Colonoscopy demonstrating edematous and dark purple-blue mucosa of the ascending colon.

**Figure 4.**
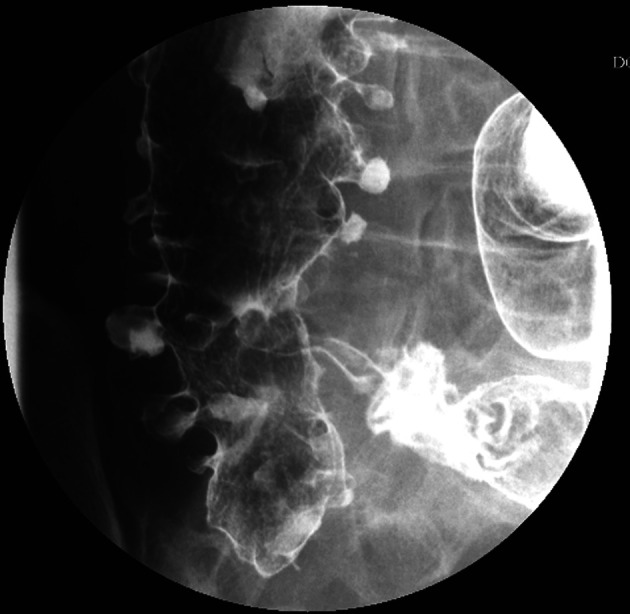
Barium enema of the colon demonstrating multiple diverticula, disappearance of the semilunar folds, irregular contours, and rigid walls of the ascending colon.

**Figure 5.**
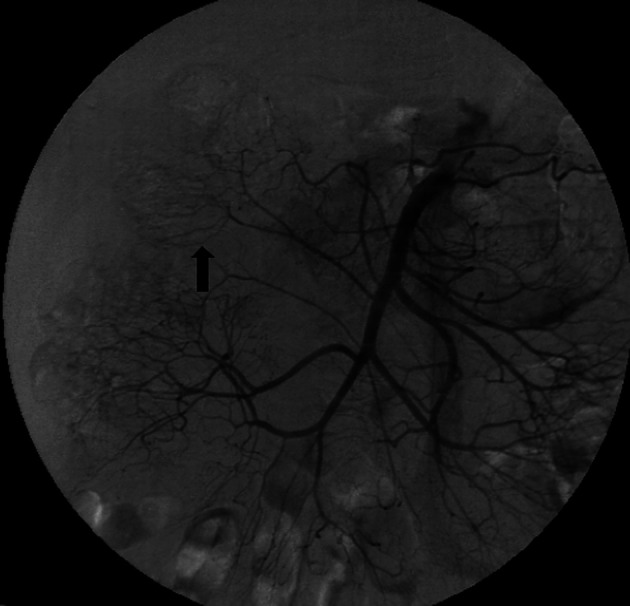
Angiography demonstrating mild tortuosity of vasa recta and marginal arteries of the ascending and transverse colons (arrow).

Idiopathic mesenteric phlebosclerosis is usually diagnosed on the basis of radiological and endoscopic studies, including abdominal plain radiography, barium enema, abdominal CT, angiography, and colonoscopy.^3,4^ On plain abdominal radiography, IMP usually shows multiple tortuous thread-like calcifications in the right-side region of the colon and may extend into the transverse colon (Figure 1). These thread-like calcifications are arranged perpendicular to the bowel wall, which suggests vascular calcifications. Although plain abdominal radiography has generally been used in patients presenting various abdominal symptoms, the fine-branching calcifications of the vasculature can be easily misdiagnosed by clinicians. Abdominal CT can be a valuable diagnostic modality and is superior to invasive angiography for IMP. It can anatomically and non-invasively depict a well-thickened colonic wall and venous calcification, the precise location, the extent of calcifications, other concomitant abnormalities, complications in the intra-abdominal vasculature, and visceral organs (Figure 2A and B). On colonoscopy, an IMP may show a dark purple edematous mucosa,narrowing, erosions, and ulcerations in the involved colon or terminal ileum due to venous congestion (Figure 3). On barium enema, an IMP presents as a narrowing and thumbprints in the right hemi-colon (Figure 4). On angiography, IMP may be observed as a dilatation in the veins along the vasa recta in the venous phase (Figure 5). The present study diagnosed patients with IMP based on the characteristic pattern of the disease on plain abdominal radiography and abdominal CT. Accordingly, 88.8% and 94.4% of the patients underwent plain abdominal radiography and abdominal CT, respectively. Colonoscopy was performed in 33.3% of all patients. However, barium enema and angiography were rarely performed in our patients for 2 reasons. First, clinicians in Taiwan lack the understanding and experience regarding this disease. Second, this disease usually presents with an acute abdomen, leading to inadequate time for further survey.

Surgical resection of the involved bowel segments has been the primary treatment approach suggested in previous studies.^8,21^ Iwashita et al^4^ who reported a case series on patients with IMP, found that 62% of all patients underwent subtotal colectomy. However, more studies on the conservative treatment of patients with IMP have emerged. Notably, Saito et al^31^ reported a case of IMP with characteristic deep circumferential ulcerations, which eventually resolved after 7 months of conservative treatment. Siao et al^12^ also reported a case of IMP successfully treated with conservative treatment. Moreover, a study by Chen et al^15^ concluded that majority of patients with IMP (88%) accepted conservative treatment, with only 3 patients undergoing colectomy. Similarly, the results of the present study showed that 91.6% of all patients with IMP accepted conservative treatment with favorable outcomes, except for the 3 who died of sepsis and respiratory failure (not directly related to this disease). No recurrence had been noted after the follow-up period. By contrast, only 8.3% of our patients underwent emergency total or subtotal colectomy given the disease severity on presentation. Indeed, deciding on whether conservative or surgical treatment should be provided to patients with IMP in clinical practice remains difficult. Lin et al^32^, who used CT scores to compare surgically and non-surgically treated patients with IMP, concluded that surgery group had significantly more colonic segments with mesenteric venous calcification and higher total calcification scores compared to the non-surgical group (4.33 vs. 2.96, *P* = .003 and 15.00 vs. 8.96, *P* < .001). Thus, conservative treatment may be prescribed for symptomatic patients with IMP determined using the CT score proposed by Lin et al.^32^

The primary limitation of the present study is our relatively small number of cases given the infrequent occurrence of IMP. Therefore, future case studies are needed to clarify the clinicopathological features of this condition.

## Conclusions

The findings pressed herein suggested that IMP is a considerably rare disease in Taiwan with distinctive imaging features that can be used for definitive diagnosis. Moreover, our findings showed that plain abdominal radiography and abdominal CT can be useful modalities in diagnosing this disease. Although no definite consensus has yet been established for the treatment of IMP, our experiences suggest that conservative treatment may be adequate for treating this disease, with surgery potentially being required in patients with ongoing severe abdominal symptoms. Thus, clinicians should consider the possibility of IMP among elderly patients presenting with acute abdomen.

## Figures and Tables

**Table 1.** Clinical Characteristics of Patients with Idiopathic Mesenteric Phlebosclerosis (n = 36)

Patient Characteristics	No. (%) of Patients
Mean age (years) (range)	61.6 (43-85)
Sex	
Female	18 (50)
Male	18 (50)
Symptom	26
Abdominal pain	22 (61.1)
Diarrhea	4 (11.1)
Fever	3 (8.3)
Bleeding	1 (2.7)
No symptoms	10 (27.7)
Comorbid disease	30
Cancer (including TCC, RCC, HCC, hepatic angiosarcoma, and gastric cancer)	18 (50)
Renal disease (including CKD and ESRD)	16 (44.4)
Liver disease (including liver cirrhosis and chronic hepatitis)	14 (38.8)
Cardiovascular disease (including CAD, HTN, heart failure, valvular heart disease and stroke)	13 (36.1)
DM	3 (8.3)
No major systemic disease	6 (16.7)
History of Chinese herbal medicines	13 (36.1)

CKD, chronic kidney disease; CAD, coronary artery disease; ESRD, end-stage renal disease; DM, diabetes mellitus; HCC, hepatocellular carcinoma; HTN, hypertension; RCC, renal cell carcinoma; TCC, transitional cell carcinoma.

**Table 2.** Diagnostic Modalities, Involved Bowel Segments, Treatment Methods, and Outcomes of Patients with Idiopathic Mesenteric Phlebosclerosis (n = 36)

Findings	No. (%) of Patients
Diagnostic modalities	
Abdominal computed tomography scan	34 (94.4)
Plain abdominal radiography	32 (88.8)
Colonoscopy	12 (33.3)
Barium enema	3 (8.3)
Angiography	1 (2.7)
Involved segments of the bowel	
Ascending colon	36 (100)
Transverse colon	23 (63.8)
Descending colon	3 (8.3)
Cecum	1 (2.7)
Terminal ileum	1 (2.7)
Treatment methods	
Conservative treatment	33 (91.7)
Surgery	3 (8.3)
Clinical outcomes	
Survival	33 (91.7)
Mortality*	3 (8.3)

*Not related to this disease (2 patients died of septic shock; 1 patient died of respiratory failure).
